# Characterization of the Key Compounds of Bell Pepper by Spectrophotometry and Gas Chromatography on the Effects of Induced Stress on the Concentration of Secondary Metabolite

**DOI:** 10.3390/molecules28093830

**Published:** 2023-04-30

**Authors:** Sandra N. Jimenez-García, Lina Garcia-Mier, Xóchitl S. Ramirez-Gomez, Ramon G. Guevara-Gonzalez, Humberto Aguirre-Becerra, Alexandro Escobar-Ortiz, Luis M. Contreras-Medina, Juan F. Garcia-Trejo, Moises A. Vazquez-Cruz, Ana A. Feregrino-Perez

**Affiliations:** 1 Division de Ciencias de la Salud e Ingeniería, Campus Celaya-Salvatierra, C.A. Enfermedades no Transmisibles, Universidad de Guanajuato, Av. Ing. Javier Barros Sierra No. 201 Esq. Baja California, Ejido de Santa Maria del Refugio Celaya, Guanajuato 8140, Mexico; 2 Departamento de Ciencias de la Salud, Universidad del Valle de México, Campus Querétaro, Blvd, Juriquilla No. 1000 A, Delegación Santa Rosa Jáuregui, Santiago de Querétaro, Querétaro 76230, Mexico; 3 Division de Estudios de Posgrado, C.A. Bioingeniería Básica y Aplicada, Facultad de Ingeniería, Universidad Autónoma de Querétaro, C.U. Cerro de las Campanas S/N, Colonia Las Campanas, Santiago de Querétaro, Querétaro 76010, Mexico; 4 Facultad de Química, Universidad Autónoma de Querétaro, C.U. Cerro de las Campanas S/N, Colonia Las Campanas, Santiago de Querétaro, Querétaro 76010, Mexico; alexandro.escobarorti@outlookl.com; 5 Departamento de Investigación y Desarrollo, Koppert Mexico, Circuito el Marques Nte. 82, Parque industrial El Marqués, Santiago de Querétaro, Querétaro 76246, Mexico; mvazquez@koppert.com.mx

**Keywords:** sweet pepper, elicitor, salicylic acid, hydrogen peroxide, electrical conductivity, bioactive compounds, functional food, metabolomics’

## Abstract

Sweet peppers are consumed worldwide, and traditional uses have sparked interest in their applications as dietary antioxidants, which can be enhanced in plants using elicitors. These are endowed with phytochemicals with potential health benefits such as antioxidants, bioavailability, and bioaccessibility. The trend in metabolomics shows us chemical fingerprints linking metabolomics, innovative analytical form, and bioinformatics tools. The objective was to evaluate the impact of multiple stress interactions, elicitor concentrations, and electrical conductivity on the concentration of secondary metabolites to relate their response to metabolic pathways through the foliar application of a cocktail of said elicitors in pepper crops under greenhouse conditions. The extracts were analyzed by spectrophotometry and gas chromatography, and it was shown that the PCA analysis identified phenolic compounds and low molecular weight metabolites, confirming this as a metabolomic fingerprint in the hierarchical analysis. These compounds were also integrated by simultaneous gene and metabolite simulants to obtain effect information on different metabolic pathways. Showing changes in metabolite levels at T6 (36 mM H_2_O_2_ and 3.6 dS/m) and T7 (0.1 mM SA and 3.6 dS/m) but showing statistically significant changes at T5 (3.6 dS/m) and T8 (0.1 mM SA, 36 mM H_2_O_2_, and 3.6 dS/m) compared to T1 (32 dS/m) or control. Six pathways changed significantly (*p* < 0.05) in stress-induced treatments: aminoacyl t-RNA and valine-leucine-isoleucine biosynthesis, and alanine-aspartate-glutamate metabolism, glycoxylate-dicarboxylate cycle, arginine-proline, and citrate. This research provided a complete profile for the characterization of metabolomic fingerprint of bell pepper under multiple stress conditions.

## 1. Introduction

One of the most important crops worldwide is Pepper (*Capsicum* spp.). Pepper fruit crops reached around 28 million tonnes of at least 2 million ha [1]. The fruits have been used as a coloring in the food industry and gastronomy and their traditional uses are fresh or cooked vegetables, condiments, or spice. The source of health-related metabolites include pepper, with increased metabolites, such as ascorbic acid, and phytochemical compounds (carotenoids, tocopherols, flavonoids and capsaicinoids) [2,3], these compounds are recognized as secondary metabolites. Recently, Wahyuni, et al. [4] presented that different Capsicum with the application of elicitors shows ability of enhance the morphological characters, and concentrations and types of metabolites with effects on health. These secondary metabolites are involved in various metabolic pathways of the plant, as well as in processes such as growth and reproduction, and also as a defense mechanism against biotic or abiotic stress [5,6,7]; Therefore, treatment with specific chemical, natural or enzymatic compounds can improve the production of secondary metabolites, called elicitors, characterized by behave as a substance that, Initiates or enhances the biosynthesis of specific compounds, when inserted in small amounts of concentrations. To a living system, [8,9,10,11]. Salicylic acid (SA) has presented a remarkable capacity as an elicitor in plants related to the potencies of phenolic compound. It occurs naturally in plants in small amounts. It participates in the stomata regulation system, responding rapidly to environment changes, nutrient uptake, biological and enzymatic activity, and exogenously sprayed SA interact with stress signaling mechanisms [12,13]. Catalase and ascorbate peroxidase are the main enzymes involved in the removal of H_2_O_2_ in plants, and SA can; inhibit their activities; this inhibition leads to a local increase in H_2_O_2_ levels, which enhances adaptive responses in plants, such as the growth response to various stimuli such phenylalanine ammonia-lyase (PAL), is the first enzyme and committed in the phenolic metabolic pathway [9,13,14]. Based on the critical role of H_2_O_2_ in stress signaling mechanisms and because this molecule is comparatively stable, it can be considered an elicitor [15,16]. Salinity level used to nutrient solutions for tomato cultivar in soilless systems, it is so in the range of 25 mM (closed irrigation system) to 40–75 mM (open irrigation system) overall ion concentration (equal to an EC of 1.6–5.0 mS.cm^−1^). Greenhouse growers maintain a balance between the vegetative and generative part of a crop plant to control water range, if they modify the electrical conductivity (EC). Cell walls in plants are strengthened applying high EC during the early phase of establishment of the plants. However, optimum yield and quality occasionally differ in the requirements for different ECs of the nutrient solution and depend on the relations between intercrops relationships, environmental condition, and cultural practices. However, commercial yield and fruit size are affected, by high electrical conductivity, improving their quality and sensory profile. On the other hand, the nutraceutical properties of crops have been the subject ofsome types of research related to the influence of salinity and change in the profile of secondary metabolites of crops [13,17]. Metabolites are essential elements at the biological levels and the mechanism in cells at the metabolite level. Metabolomics profiles show that metabolites and low molecules weight compounds interact with different types of genes and proteins that leave behind specific cellular processes, evidencing the phenotypic compared to genomic analyses [18,19]. Metabolomics has helped to find biomarkers and metabolomics fingerprints in humans and crops, which analyzes each meabolote thet makes up a biological sample [20,21,22,23]. Recently, high-pressure liquid chromatography (HPLC) and gas chromatography-mass spectrometry (GC-MS) has been used to recognize a set of metabolites well-known as metabolomics. The identity of the metabolites can be simultaneously detected in a biological sample. This assay may help identify changes in secondary metabolites profile to identify compounds correlated with different traits of interest such as pungency, color, or flavor in fruits. Metabolomics-assisted has focused on applying metabolomics strategies and their integration with other high-throughput technologies of metabolite-based quality traits [24,25,26]. In this regard, this work aimed to assess the effect of multiples-stress interactions of different concentrations of elicitors (SA and H_2_O_2_) and three different levels of electrical conductivity on the concentration of secondary metabolites and correlate their changes with pathways topology in each on a pepper crop under greenhouse conditions to predict plant responses to future climate changes. Since several distinct environmental stimuli can affect plant development in combination, many stress-responsive signal pathways must be integrated to adapt resource allocation between defense, growth, and reproduction most efficiently.

## 2. Results

### 2.1. Metabolomic Profiling of Sweet Pepper Red

The data collected for phenolic compounds, carotenoids, vitamins, aminoacids, organic acids, fatty acids and phytosterols in the different treatments of sweet pepper were analyzed. Metabolic profiling nowadays appears as central tools in chemical plants for screening the pool of bioactive compounds and elucidate regulative principles and pathways. Phenolic compounds are an efficient way to describe interpretation of plant protection metabolites might wholly require monitoring metabolites in the plant’s tissue or on its shallow. It needs to be mentioned that the relationship between sweet pepper and metabolic profile is that flavor mostly comes from the ratio of reducing sugars to organic acids. In this regard, carotenoids contribute to the particular Flavor, aroma and color of sweet pepper. Fatty acids and phytosterols are also related to biosynthesis of metabolic pathways.

### 2.2. The Phenolic Profile of Sweet Pepper

In the Table 1 showed that treatments only affected bioactive compounds related to phenolic compounds, carotenoids, vitamins, aminoacids, organic acids, fatty acids and phytosterols. Table 1 shows the F-ratios and *p*-values acquired in this statistical assessment. Whenever, significant differences among means were sensed, multiple range tests (MRT) were approved to evaluate them. The phenolic profile of sweet pepper treated with salicylic acid (SA) (0, 0.01, and 0.1 mM), hydrogen peroxide (H_2_O_2_) (0, 18 and 36 mM) and Electrical conductivity (EC) (3.2, 3.4 and 3.6 dS/m) are shown in (Table 1). For phenolic compounds the different elicitor treatments only showed significant differences for the compounds identified showed an increase in the chlorogenic and caffeic acid, and resveratrol (F = 214.88; *p* = 0.000), (F = 10.439; *p* = 0.001), and (*p* = 8.474; F = 0.002) respectively. Treatment 6 increased the concentration chlorogenic and caffeic acid, treatments 2, 3, 7 showed significant differences in the concentration in resveratrol (Table 1) found in treatments treated with stress-induced for CE compared to the control.

On the other hand, the carotenoids showed an increase in the majority lutein (F = 7.965; *p* = 0.000), lycopene (F = 5.178; *p* = 0.000), and β-carotene (F = 3.256; *p* = 0.005). Treatment 8 increased the concentration lutein, lycopene and β-carotene. This treatment was treated with cocktail of stress-induced showed an increase in most of these compounds compared to control treatment Table 1. Also, ascorbic acid showed in the treatments affected for stress-induced significant differences (F = 0.731; *p* = 0.664). Table 1 identifies low molecular weight polar compounds obtained by GC–MS analysis, such as organic acids, amino acids and phytosterols. stress-induced treatment led to the presence of metabolites not detected in the control; for instance, six amino acids, nine organic acid and α-tocopherol were detected in sweet pepper obtained from plants treated with stress-induced, but not in the control sample. Although the point of fact that elicitation may significantly lofty the production of secondary metabolites. Elicitation with H_2_O_2_ caused a significant decline in amino acid content. The uppermost levels of amino acids were determined in treatment 5 elicited with EC 3.6 dS/m, increasing by 8 amino acid compared to control. However, in the treatment 2 of 36 mM H_2_O_2_ and EC 3.2 the reduction was much higher 2 amino acid in comparison to control. Our previous studies showed that H_2_O_2_ applied as cocktail of SA and EC in surface of a sweet pepper increased stress tolerance effectively. This caused a similar response in organic acids.

### 2.3. Comparative Profiling of Metabolites in the Different Treatments under Elicitor Application and Electric Conductivity Levels by PCA and HCA

**Figure 1:**
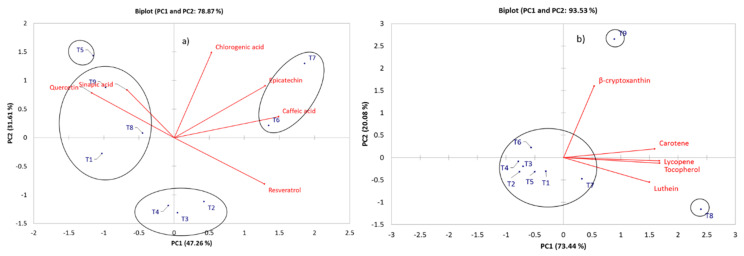
Principal component analysis (PCA) of (**a**) phenolic compounds and (**b**) carotenoids in pepper samples. Hierarchical clustering analysis (HCA) allowed identification of clusters.

Metabolic profiling of the pepper samples allowed identifying 43 compounds belonging to 6 different chemical groups by HPLC and GC-MS. Metabolites of the pepper fruits were first analyzed by PCA separating compounds by chemical groups. The analysis for phenolic compounds is shown in (Figure 1a), the first two principal components (PC) described 78.87% of the variation among treatments. PC1 described 47.26% of variation, whereas PC2 described 31.61%. Epichatechin, caffeic acid, quercetin and resveratrol mainly described the variation in PC1. PC2 was described by chlorogenic acid. HCA grouped treatments according to similarities in metabolite profile in 4 clusters, treatments 1, 8 y 9 were grouped by their similitudes in quercetin and sinapic acid, while treatments 6 and 7 were characterized by epicatechin, caffeic acid, and chlorogenic acid. Resveratrol was characteristic for treatments 2, 3, and 4.

In the carotenoid analysis, the PCA revealed that the first two PCs described 93.53% of the variation (Figure 1b). According to the carotenoid profile, PC1 (73.44%) separated the lutein, tocopherol, lycopene and carotene for the treatments. These compounds were characteristic for treatment 7 and 8, while PC2 (20.08%) described treatment 9 in terms of β-cryptoxanthin. 

**Figure 2:**
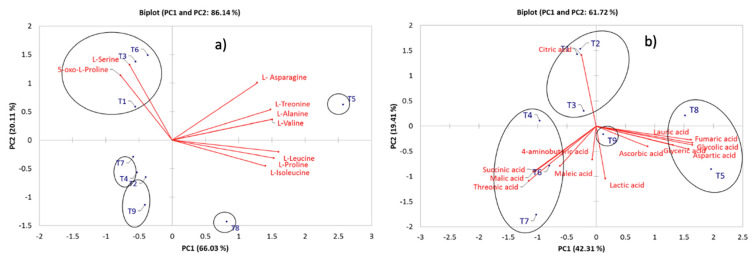
Principal component analysis (PCA) for (**a**) aminoacids, (**b**) organic acids for pepper samples. Clusters identification performed by HCA.

PCA for the aminoacids profile showed an 86.14% of the variation described by the first two PCs. L-alanine, L-valine, L-leucine, L-isoleucine, L-proline, L-treonine, and L-asparagine described PC1 (66.03%). PC2 (20.11%) was described for L-serine and 5-oxo-L-proline. According to HCA, 5 clusters were identified among treatments. Treatments 1, 3 and 6 are well described by L-serine and 5-oxo-L-proline, for treatment 5 the more characteristic compounds were L-asparagine, L-treonine, L-alanine, and L-valine. For treatment 8 are well described by L-leucine, L-proline, and L-isoleucine (Figure 2c).

The PCA for the organic acids identified by GC-MS revealed a 61.72% of the total variation explained by PC1 and PC2. PC1 (42.31%) was characterized by glycolic, succinic, glyceric, fumaric, malic, aspartic, threonic, and lauric acids. PC2 (19.41%) was described by citric acid. After HCA 3 clusters were generated, treatments 1, 2 and 3 can be well described by citric acid in PC2. Treatments 8 and 5 are described by lauric, glyceric, fumaric, glycolic, aspartic and ascorbic acids. Treatments 4, 6 and 7 were well described in terms of succinic, malic, threonic, maleic and 4-aminobutyric acids (Figure 2d). 

**Figure 3:**
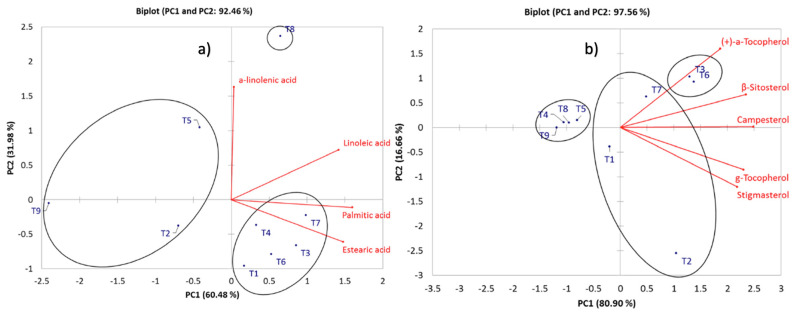
Principal component analysis (PCA) for (**a**) fatty acids and (**b**) phytosterols for pepper samples. Clusters identification performed by HCA.

The PCA for the fatty acids profile on sweet pepper samples explained a 92.46% of the total variation with the first two PCs. Palmitic, linoleic, and stearic acids described PC1 (60.48%). In comparison, PC2 (31.98%) was described by a-linoleic acid (Figure 3e). According to HCA three different clusters were generated, treatments 1, 3, 4, 6, and 7 were grouped and described by stearic and palmitic acids. Treatment 8 was well correlated with a-linoleic ad linoleic acids. The last group of compounds was the phytosterols, PCs 1 and 2 explained a 97.56% of the total variation, contributing PC1 with 80.90% and PC2 with 16.66%. All the phytosterols were characteristic for the PC1. (+)-α-tocopherol was well correlated with treatments 3, 6 and 7 (Figure 3f).

### 2.4. Metabolic Profiles of Sweet Pepper (C. annum L.) Stress-Induced

**Figure 4:**
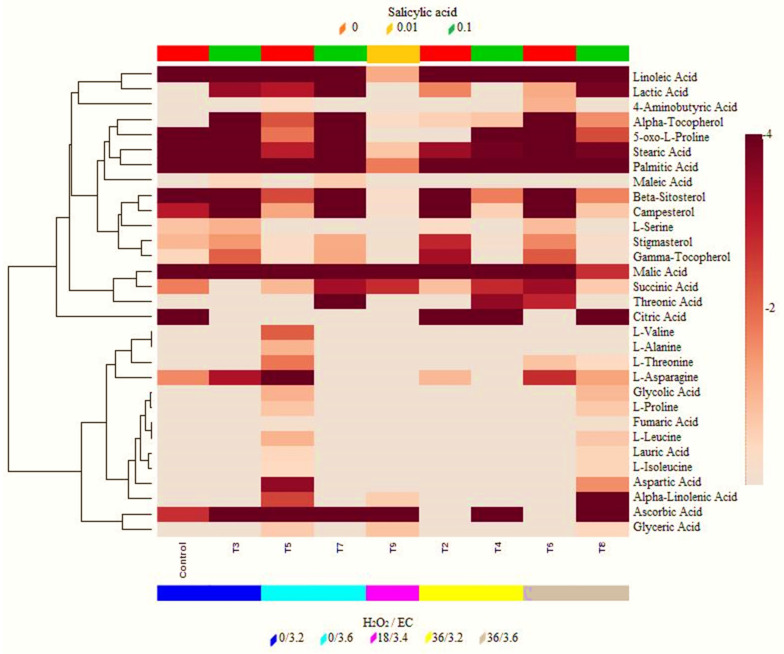
Heat map of 32 metabolites accumulated in sweet pepper (*C. annuum* L.). The lower bars indicate the sample classes (control, T2, T3, T4, T5, T6, T7, T8, T9). The columns represent stress-induced (SA and H_2_O_2_/EC), and the rows refer to distinct metabolites. The values of the metabolite’s concentration have depicted with an encoded-color matrix from the dark/light in every genotype, which has been log_2-_ changed and mean-centered.

The commercial NIST library showed to a comparison the spectra of the treatments recognized thirty-one metabolites. A plotting map of all recognized metabolites is presented in Figure 4 to present the differences between the stress-induced and control treatments. The metabolite levels enhanced moderately in the stress-induced treatments compared with the control treatment. Eighteen metabolites were disturbed by stress-induced treatments, respectively. The stress-induced treatments significantly increased six metabolites, namely campesterol, stigmasterol, (-tocopherol, succinic acid, asparagine, and ascorbic acid. In addition to glyceric, fumaric, maleic acid and isoleucine any and every of another one metabolites showed higher changes in the stress-induced treatments. β-sitosterol was decreased in treatment 4, 5, 8 and linoleic acid was decreased more by treatment 9 than other treatments. The affinity of three more metabolites showed a trend: the levels of 4-amonobutyric acid, alanine, and valine were not present in most treatments but increased in treatment 5. The metabolites in the bottom group were present at diverse comparative abundances in everything treatments verified. Their exclusion pattern in the treatment 5, 8 were not specific, since genotypes with upper and lower affluence of these metabolites were present lower borderline detection. Metabolites in top cluster collected at relatively upper abundance in the sweet pepper fruits. treatment 2, 3, 6, 7, were specific with higher abundance of these metabolites, some of this group were putatively identified as steroidal glycosides detected anteriorly in *C. annuum var. acuminatum* seeds with a antimicrobial activity opposite yeast and fungi [27,28]. 

**Figure 5:**
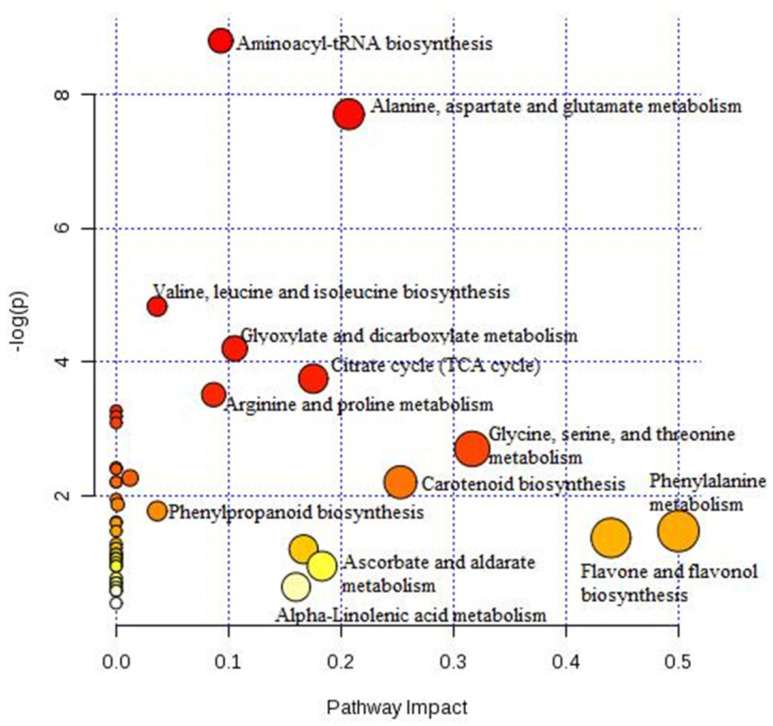
Graphic of the metabolome subsequent the metabolite pathway plotting of the impacted metabolites recognized after result stress-induced in sweet pepper (*C. annuum* L.). A color coded matrix indicates concentration values of the metabolic pathway and result of each metabolite, which has been log_2-_ changed and mean-centered. (Color figure online). The investigation was achieved using the MetaboAnalyst software. 5.0.

It was used MetaboAnalyst’s to recognize and analyze an improved planning of metabolomics studies and biological interpretation in pathway. The eighteen metabolites predominated to identify the expression of various pathways during multiple stress. Some of these compounds were responsible for activating multiple pathways. On the other hand, intermediates were detected, such as fatty acids (linoleic acid and palmitic acid), organic acids (malic acid and succinic acid), phytosterols (Campesterol and sitosterol), phenolic compounds (epicatechin and caffeic acid). The detailed diagram indicates the metabolites that have the greatest effect on the metabolic pathways shown in Figure 5. Thus, six pathways were significantly (*p* < 0.05) confounded in the stress-induced treatments: aminoacyl biosynthesis t-RNA, alanine, aspartate and glutamate, valine, leucine, and isoleucine biosynthesis, glyoxylate and dicarboxylate metabolism, citrate cycle (TCA cycle) and arginine and proline metabolism. High-quality KEGG metabolic pathways were established and used by researchers to find the most significant pathways. Compared to control, flavonoids and phenolic compounds show activation and upregulation in all stressed groups.

## 3. Discussion

The main compounds identified in the sweet pepper were phenolic acids, flavan-3-ols, flavonols and flavonones. Vanillic increased in ginger treated with 10-3 and 10-5 M SA, but not in controls. Sinapic acid have coumaric acid as a precursor, which could be related with the stimulation of phenylalanine ammonia-lyase. Another enzyme regulated in flavonoid synthesis is chalcone synthase [29,30,31]. Studies with tomatoes and carrots using different EC with salts solutions in the irrigation system not showed difference between treatments in the concentrations of catechin, epicatechin, trans-resveratrol [32,33,34]. Therefore, this study confirms the results, in this investigation as there are no differences between treatments applied in sweet pepper by the EC. Furthermore, Medina–Juárez, et al. [35] observed in the extract, caribe and sweet pepper corresponding to the highest levels of resveratrol, epicatechin, luteolin, rutin, gallic and chlorogenic acid, also acid ascorbic (r ≥ 0.85). To compare our results we found that the treatments treated with the cocktail of elicitors have higher concentration of these compounds to those reported in sweet pepper without stress-induced. These results propose that applying cocktails elicitor, SA and H_2_O_2_, may cause oxidative stress in the sweet pepper, resulting in increased production and synthesis of different phenolic compounds. Also, the main compounds identified in the sweet pepper were Lutein, tocopherol, β-criptoxantin, licopene and β-carotene. Treatments 8 showed an increase in the majority in lutein, and β-carotene compared to treatment control. Peppers mostly contained violaxanthin, capsanthin, lutein, and β-carotene. Therefore, in the chloroplasts tended to decrease the content of the major on these compounds in all maturation time and at the same time were formed new xanthopylls to become a mono- and/or diesters with the union of a fatty acid [36]. The level of carotenoid compounds varied with different colored peppers. Yellow pepper had the uppermost level of carotenoids, although green pepper had the lowermost. Deli, et al. [37] investigated yield of carotenoids (β-cryptoxanthin, α- and β-carotene) peaked and then decreased in yellow pepper (*C. annuum lycopersiciforme flavum*) during the maturation. On the other hand, they also identified lycopene, zeaxanthin, lutein, and capsanthin as secondary metabolites used in chronic-degenerative diseases [38,39,40]. The results of these research carotenoid concentrations are higher than those previously reported in peppers of different colors optimal ripeness. Our results were found that the treatment 8 treated with the cocktail of elicitors have higher concentration of these compounds to those reported in sweet pepper. These results suggest that applying cocktails elicitor, SA and H_2_O_2_ and EC, may cause oxidative stress in the sweet pepper, resulting in increased production synergism and higher synthesis of carotenoids. The ascorbic acid contents of sweet pepper cultivars resemble other pepper crops previously studied by Howard, et al. [41] and Simonne, et al. [42]. Nevertheless, other research’s described higher ascorbic acid concentrations in upper levels with the current results without stress-induced. Ascorbic acid advanced bleaching of the β-carotene emulsion [43]. Our result also presented that ascorbic acid had pro-oxidant effect. Then, we propose that the antioxidant activity in the methanol extracts ought to be the likeness of the result of phenolic compounds and the pro-oxidant result of ascorbic acid [44]. The ascorbic acid contents of the treatments were higher of 1.3 mg/g than those reported. These changes can be consistent by EC. The nutrition value can assess the content of ascorbic acid used standards US DRI which indicates the consumption of ascorbic acid are 75 mg/day for males and 90 mg/day for females (IOM, 2000). Taking into account these values, to consume 17–72% for males (ages 19–50 years), and 20–86% for female (ages 19–50 years) of ascorbic acid per day, they require consumption of 100 g of mature fruit and this depends on the type of crop. On the other hand, sweet peppers have improved stress tolerance to sprinkled as cocktail of SA/H_2_O_2_ and EC about to superficies of sweet pepper. These cocktails also have increased the amount of organic acids. On the other hand, HancockandSahl [45] showed that minimum concentrations of the H_2_O_2_ molecule have not shown the receptor proteins that intervene in the detection of the same. They evidenced that amino acid perception was controlled by other characteristics of the proteins having active thiol groups as redox targets. Oxidation of the thiol groups by H_2_O_2_ and other bioactive compounds, was generated by oxidants for the thiol groups [46]. Furthermore, SA is an important regulator of photosynthesis, since it increases the activity of two key enzymes in carbohydrate synthesis, RuBisCO and carbonic anhydrase [47]. Furthermore, some studies showed that phenolic acid synthesis in crops not heighten the carbohydrate concentration, since the phenolic acid pathway uses products of carbohydrate metabolism as precursors [48,49,50]. Furthermore, the amount of organic acids as palmitic, stearic, oleic, linoleic acid were increased by changes in electrical conductivity, similar results are shown by Kaewnaree, et al. [51] with seed sweet pepper.

This study discovered that the metabolite levels were significantly altered in the treatment 5, 6, 7, 8 with the control. The stress-induced radiation increased the secondary metabolites such as phytosterols, fatty and organic acids, amino acids. Rahimi, et al. [52] investigated the increase to the essential oil and quality of cumin (*Cuminum cyminum* L.) were caused for to induced-stress of salicylic acid and methyl jasmonate, which main effect was an increase by the application of SA. Moreover, the influence of increasing doses of salicylic acid (SA) showed growth-promoting effect with decrease in tissue water content, chlorophylls and soluble proteins in *Matricaria chamomilla* plants. As well as decrease in chlorogenic acid [53,54]. On the other hand, Hao, et al. [55] investigated the effect of SA and H_2_O_2_ in *Salvia miltiorrhiza* and yield rosmarinic acid. These researches showed that yield in H_2_O_2_ production, induced by stress of SA increased rosmarinic acid and phenylalanine ammonia-lyase (PAL) activity. Other investigation showed that H_2_O_2_ is a signaling molecule that allow in develop signals on different plant metabolic pathways [56] and increasing to activity other molecules of key signaling (Ca^2+^, SA, ABA, JA, ethylene, NO) of plants [57]. Also, the catabolism of amino acids and fatty acid elongation in the plant produce the acyl residues of capsaicinoids [58,59]. P-coumaric acid is the key signal in the capsaicin pathway and likewise is important in synthesis of lignins, flavonoids, hydroxycinnamic polyamides and pigments [60]. Coumaroyl, feruoyl and cinnamaldehydes were reduce by cinnamoyl CoA and sinapoyl-CoA respectively; therefore, the phenylpropanoid pathway is to point of signaling by lignin biosynthesis, being of great importance in detection of capsaicinoid levels [61,62]. In this study, fatty acids symbolized the main group of differential metabolites. The plant responds to pathogen attack high salinity, drought and low temperature, producing higher fatty acid [63,64,65]. On the other hand, interact synergistically lipids with tannins in barley were try in bacterial and fungal pathogens inhibiting the growth [64]. Likewise in radish, maize, and grape [64,66]. Moreover, the lipids could inhibit sensitive pathogens, because were scattered above the plant and in the vascular tissues [64,67,68]. Forefather phyto-oxylipin biosynthesis in linolenic acid al also produces indiced-stress in plants [69]. The chloroplast oleic acid levels affect systemic acquired resistance in Arabidopsis [70]. Likewise, the decrease of fungi and the mycotoxin produced by *Aspergillus* spp could to eliminated with oleic and linoleic acid [71]. Hence, Capsaicinoid pathway could increase the activity with methyl-branched fatty acid esters, this is produced in the placenta, and transported into the apoplast and later, to tissues or organs [72]. In addition stress-induced can increase/decrease to production of hydrocarbons, phytosterols, fatty and amino acids, TCA cycle intermediates. The pathway enrichment analysis showed stress-induced that increase of six pathways as such as citrate cycle, minoacyl-tRNa biosynthesis, nitrogen metabolism. The plant activity was affected by nitrogen metabolism. Also, the generation of energy is produced with nitrogen metabolism that is the point more important and the union of the metabolic pathway. Amino acids play important roles and the conditions of salt stress on sugar beets (*Beta vulgaris* L.) resulted in a quick increase in proline content. The free amino acids were moderately improved by means of stress-induced of the plant, the basic amino acids and amides were lot improved y the aspartic and glutamic acids were slightly affected of stress-induced [73]. The overall metabolite profiles (Vitamins, carotenoids, flavonoids and capsaicinoids) have greater effect by the synergistic interaction against the individual effects of each type of metabolite [4,74]. Under stress conditions less energy and nutrients are allocated to growth processes and more is invested in defense responses. This requires a fine tuning of nutrient and energy allocation between growth/reproduction and defense associated processes by complex signaling networks integrating incoming developmental and environmental signals. Since phytohormons often magnify the initial signal and trigger a new signaling event either following the same pathway or using further components of other signaling pathways [75]. Combined occurrence of abiotic and biotic stress may result in synergistic, and antagonistic or dominance interactions. The outcome will depend on the timing, nature, and severity of each stress [76]. Potentiation of health-related secondary metabolites can be caused by the stress-induced in the plants caused by the use of elicitor and biotic or abiotic stress. These metabolites are always requested by the consumer as these produce texture taste or color or raise the pungency produced by carotenoids, flavonoids or capsaicinoids. 

## 4. Materials and Methods

### 4.1. Greenhouse Climatic Conditions

This study was conducted under greenhouse conditions from August to January, using red sweet peppers (*Capsicum annuum* L) fasinato variety in a community close to Ezequiel Montes, Queretaro, and México. Plants were grown hydroponically using rock wool a substrate and fertilized by drip irrigation to provide all the nutrients to the plant. Pepper plants were distributed with planting distances of 1.5 m among rows and 0.3 cm amid plants in each row as such a final density of 2.3 plants m^−2^. Climate conditions were monitored daily; the mean values were 25 °C and 85% HR. Pepper fruit when fully ripen (surface coloration was completely red). 

### 4.2. Experimental Design 

Research design was applied a factorial complete [77]. Treatment factors in this experiment were different concentrations of salicylic acid (SA), hydrogen peroxide (H_2_O_2_) and three different electrical conductivities. 9 treatments resulted from the different of mixture of factors and their levels (Table 2) 

### 4.3. Fruit Material and Preparation

Samples of 1.5 kg of fully mature sweet pepper were collected seven weeks after fruit set. The stems were removed and sweet pepper was cut into cubes (2 cm^2^), and they were frozen in liquid nitrogen to be subsequently lyophilized. Subsequently, a lyophilizer (Labconco FreeZone 4.5 L model 230 V) was used at a temperature of −50 °C with a 0.01 atm vacuum system until the sample dried [78]. The sample was stored in 50 cm falcon tubes where the O_2_ in the headspace was eliminated to be stored in cool and dry spaces for analysis. The preparation of the sample for the analysis of phytochemical compounds will be carried out according to the methodology described by Cardador–Martínez, et al. [79], 25 mg of dry sample will be placed and 2.5 mL of methanol will be added to each sample, performing samples in triplicate. They were kept out of light and shaken for 24 h. Centrifuging at 5000 rpm/10 min/4 °C, the sediment formed at the bottom was removed, leaving the supernatant.

### 4.4. Identification of Phenolic Compounds

Phenolic compounds in the sweet pepper were determined and measured two varieties of compounds flavonoids and phenolic acids using an Agilent 1200, HPLC system, coupled with an auto-sampler and a UV/Vis detector. The separation was performed on a Sorbaz eclipse plus column (C18 and (250 × 4.6 mm, 5 μm)) reversed-phase. Samples (10 μL) were inserted with a gradient elution involved two solvents: A (formic acid 1%/water, *v*/*v*) and B (acetonitrile), at a current rate of 1 mL/min. The solvent gradient was programmed in a ratio A:B (98:2 *v*/*v*, 0 min; 68:32 *v*/*v*, 30 min; 45:55 *v*/*v*, 48 min; 5:95 *v*/*v*, 53 min; 98:2 *v*/*v*, 57 min), with 67 min of run time. The standards of the suspected phenolic acids and flavonoids used (chlorogenic, ellagic, caffeic, coumaric, sinapic and rosmarinic acids as well as gallocatechin-gallate, epicatechin, vainillin, rutin, quercetin, hesperidin and resveratrol) (Sigma-Aldrich with 99.99% purity) were measured in a range 260–320 nm.

### 4.5. Identification of Carotenoids

Carotenoids in the sweet pepper were recognized and quantified using an Agilent 1200, HPLC-DAD system, coupled with an auto-sampler and a UV/Vis detector. The separation was performed on a Sorbaz Eclipse Plus C18 column (250 × 4.6 mm, 5 μm) in reversed –phase. Samples (20 μL) were inserted with a gradient elution involved two solvents: (A) methanol and (B) acetonitrile, in a ratio (A:B) of 55:45 at a current of 1 mL/min up to 13.59 min and subsequently the 14 min to 30 min a current of 2 mL/min, a total run time of 30 min. Absorbance magnitudes were from 290–450 nm. Carotenoids were identified using standards such as (lutein, zeaxanthin, tocopherol, β-croptoxantina, lycopene and pure β-carotene) (sigma-Aldrich with 99.99% purity).

### 4.6. Identification of Ascorbic Acid

Ascorbic acid (AA) was definite in respected of the method of Ruiz–Cruz, et al. [80]. First, samples of a homogeneous peppers suspension (2 g) of 4% metaphosphoric acid (15 mL) were added. Then, the sample was sonicated/5 min (Sonicator VWR model 150 D; VWR International., West Chester, PA, USA) and centrifuged at 1610× *g*/10 min at room temperature (Centrifuge Thermo Scientific Sorvall ST16, Porton Down, UK). The sample supernatant was filtered through a polyethylene cartridge of 0.45 μm of pore size (Millipore Corp., Bedford, MA) and identified and quantified using a Agilent 1200, HPLC-DAD system, coupled with an auto-sampler and a UV/Vis detector. Ascorbic acid was scanned in reversed-phase at λ = 254 nm on a Sorbaz Eclipse Plus C18 column (250 × 4.6 mm, 5 μm). Samples (20 μL) were inserted with a gradient elution isocratic and the flow rate 1 mL/min, which was kept at 25 °C. The mobile phase was programmed of 0.2 M KH_2_PO_4_, pH 2.3 with 85% o-phosphoric acid. Ascorbic acid was prepared, identified in pepper extracts as a standard compound, and used for quantification calibration curves of pure Ascorbic acid (Sigma-Aldrich, DEU). Each extract was analyzed three times.

### 4.7. Gas-Chromatography-Mass Spectrometry (GC-MS) Analysis

Methanol extract was prepared (1 mg/mL), the methanol vaporized and subsequently was added 50 μL of derivatizing agent, BSTFA (N,O-bis [trimethylsilyl] trifluoroacetamide) + 1% TMCS (trimethylchlorosilane), and agitated for 2 min at room temperature. Finally, sample (1 μL) was inserted at a GC–MS system (Agilent GC Series 7890A and an Agilent single quadrupole MS detector Agilent model 5975C)), with electron impact model (70 eV) and the mass range at 50–700 *m*/*z*. The separation was performed on an HP-5MS capillary column (30 m × 0.25 mm i.d. × 0.25 μm) and splitless injector (at 250 °C) was used to 2.5 min/splitless time. The initial oven temperature was kept at 100 °C for 1 min and raised to 220 °C at 6 °C/min, and kept constant for 1.23 min, afterward raised to 290 °C at 10 °C/min, and raised to 310 °C at 40 °C/min, and kept constant for 7.5 min. The carrier gas (helium) flow rate was maintained at 1 mL/min. The GC–MS control and data processing was identified by comparing their mass spectra using Chem-Station (Agilent Technologies, DEU) software. Metabolite identifications was analyzed the spectral (MS) matching with commercialized libraries (NIST) based upon retention time, *m*/*z*, and percentage of spectral similarity greater than 60% and the internal standard.

### 4.8. Statistical Analysis 

All results obtained by high pressure chromatography liquid were the mean ± standard error. Furthermore, one-way ANNOVA and changes amongst processing evaluated data were evaluated by comparison of means using Tukey’s test. The level of statistical significance was considered at *p* < 0.05. In addition, principal component analysis (PCA) with an auto scaling using mean centering method and hierarchical clustering analysis (HCA) with centroid linkage clustering for the responses of the identified compounds were performed to characterize effect of treatments on metabolic profiling using XLSTAT 2014.05.03. 

### 4.9. Hierarchical Clustering and Pathway Enrichment Analysis

Hierarchical clustering was achieved on the log-transformed standardized data with each sample as a separate cluster and then combining them until all samples belong to one cluster. Each standardized value to enable log change was added a small value (unity) with a level of statistical significance *p* < 0.05. Hierarchical clustering was integrated by the log-transformed data acquired of the median-centered per metabolite for better visualization. The similarity metric was evaluated by Pearson’s correlation and a clustering method ward’s linkage. The metabolites’ heat maps were carried out using color scheme light/dark. These color scheme showed which metabolic pathways are influenced per stress-induced (SA and H_2_O_2_/EC), these results were analyzed and mapped using the MetaboAnalyst 5.0 software [81]. The identified metabolites were compared to Arabidopsis thaliana (*Thale cress*) pathway catalogue, since it is one of the most important systems for the study of many aspects of the biology of plants and to prevent the great economic losses that cause diseases, they have been applied to the study of the mechanisms that regulate resistance in wheat, potato or tomato and pepper; were employed in over representation analysis the hypergeometric test, and the out-degree centrality algorithms. The P-value was adjusted to improve or adapt pathway and an impact factor from the pathway physical map for each analysis.

## 5. Conclusions

In conclusion, some plant responses to unfavorable growth conditions are specific. In contrast, others are general responses providing tolerance to several stress conditions as stress response mechanisms include changes in some metabolic pathways and induction of antioxidant defense pathways against reactive oxygen species. Generally, it is assumed that stress sensing acts at multidimensional levels integrating several sensing mechanisms in specific stress conditions. Whence, to improve the understanding of plant responses to combined stress situations. We applied the system analyzing (PCA) of three stress factors and the overall metabolite profiles in plants subjected to the triple stress of SA, H_2_O_2_ and EC. One of the key findings of this work was that increase of secondary metabolites. For example, the interaction of SA and H_2_O_2_ in treatments 2, 3, 6 and 7 shows a significant effect on producing phenolic compounds. Related to carotenoids, treatment 8 promoted a gain in the concentration of these compounds. Otherwise, treatments 5 and 8 also showed a significant effect on the concentration of aminoacids, fatty acids, and phytosterols compared to the control treatment. The latter compounds are derived mainly from aminoacyl-tRNA biosynthesis; valine, leucine and isoleucine biosynthesis; alanine; aspartate and glutamate; glyoxylate; and dicarboxylate metabolism.

In conclusion elicitors can promote important changes in secondary metabolites profile, aminoacids, and fatty acids which can impact sensory traits affecting the quality of pepper due to their interactions with environment conditions. Marked disturbances in the metabolites in sweet pepper a factor of defense against insects or microorganisms, also this can change the resistance to various stresses during the grown period. In abstract, the results of our investigation propose that the wide biochemical difference in sweet pepper is under metabolic control. Combining metabolomics and the biochemical data sets will help growers to increase new pepper varieties that unite a desirable taste and nutritional profile with resistance to important chronic degenerative illnesses. Likewise, the responses of biotic and abiotic interactions could identify gene expression patterns, which are unique to combined stress situations and might give possible explanations for a changed pathogen behavior, and the importance to study biotic and abiotic stress combinations to predict plant responses to future climate changes.

## 6. Patents

This research did not generate any patent during its development.

## Figures and Tables

**Table 1:** Chromatographic profile of low molecular weight, and secondary metabolites identified in sweet pepper (*Capsicum annuum* L.).

	Treatments										
Compound (Groups)	1	2	3	4	5	6	7	8	9	F (F-Ratio)	*p* (*p*-Values)
Phenolic compounds											
*Chlorogenic acid*	-	-	-	-	2746.79 a	2414.41 a	4229.29 b	2376.57 a	2538.03 a	214.88	0.000
*Epicatechin*	-	-	-	-	577.30 a	3989.41 a	8896.08 b	-	1052.83 a	17.69	0.000
*Caffeic acid*	-	717.21ab	-	-	-	1353.34 b	1239.91 b	-	-	10.439	0.001
*Sinapic acid*	-	-	-	-	98.59 a	-	-	-	-	2.326	0.115
*Quercetin*	175.02 a	71.09 a	73.53 a	77.32 a	159.95 a	61.04 a	82.79 a	85.45 a	229.73 a	2.772	0.075
*Resveratrol*	-	180.42 b	197.42 b	161.74 ab	-	158.13 ab	177.85 b	-	-	8.474	0.002
Carotenoids											
*Luthein*	0.0017 ab	0.0015 ab	0.0015 ab	0.0013 ab	0.0016 ab	0.0009 a	0.0019 b	0.0029 c	0.0017 ab	7.965	0.000
*Tocopherol*	0.0075 b	0.0072 b	0.0074 b	0.0074 b	0.0082 b	0.0081 b	0.0097 b	0.0173 a	0.0114 b	7.214	0.000
*β-cryptoxanthin*	0.0012 a	0.0012 a	0.0012 a	0.0012 a	0.0012 a	0.0012 a	0.0012 a	0.0012 a	0.0015 a	1.074	0.398
*Lycopene*	0.0060 a	0.0057a	0.0018 a	0.0027 a	0.0048 a	0.0057 a	0.0115 ab	0.0227 b	0.0128 ab	5.178	0.000
*Carotene*	0.0149 ab	0.0102a	0.0133 ab	0.0129 a	0.0127 a	0.0160 ab	0.0164 ab	0.0230 b	0.0190 ab	3.256	0.005
Vitamins											
*Ascorbic acid*	0.84 a	1.12 b	1.09 b	0.85 a	1.13 b	0.96 ab	1.12 b	1.31 c	0.98 ab	0.731	0.664
Aminoacids											
*L-Alanine*	-	-	-	-	1.087 a	-	-		-	0.000	0.000
*L-Valine*	-	-	-	-	2.167 a	-	-		-	0.000	0.000
*L-Leucine*	-	-	-	-	1.07 a	-	-	0.771 a	-	0.021	0.281
*L-Isoleucine*	-	-	-	-	0.441 a	-	-	0.553 a	-	0.008	0.000
*L-Proline*	-	-	-	-	0.796 a	-	-	0.731 a	-	0.724	0.001
*L-Serine*	0.84 b	0.317 a	1.094 c	-		0.908 b			-	0.963	0.000
*L-Treonine*	-	-	-	-	1.845 c	0.834 b		0.454 a	-	0.384	0.000
*5-oxo-L-Proline*	10.996 b		16.181 bc	10.517 b	1.887 a	18.465 c	15.526 bc	2.399 a	-	0.551	0.391
*L- Asparagine*	1.63 c	0.982 bc	3.181 a		6.612 b	2.757 a		1.325 a	-	1.522	0.000
Organic acids											
*Lactic acid*	-	1.709 a	3.423 ab		3.083 ab	1.207 a	7.663 b	3.843 a	-	1.083	0.000
*Glycolic acid*	-	-	-	-	1.103 a	-	-	0.977 a	-	0.774	0.000
*Succinic acid*	1.751 b	0.893 ab		2.827 a	0.988 ab	3.366 a	3.284 a	0.691 a	2.753 a	1.691	0.000
*Glyceric acid*	-	-	-	-	0.696 a	-	-	0.487 a	0.838 a	0.478	0.000
*Fumaric acid*	-	-	-	-	0.184 a	-	-	0.136 a	-	0.096	0.291
*Maleic acid*	-	-	0.529 a	-	-	-	0.671 a	-	-	0.4523	0.000
*Malic acid*	4.103 a	4.6 a	4.59 a	13.577 b	5.627 a	9.011 ab	10.592 ab	2.722 a	8.48 ab	1.527	0.002
*Aspartic acid*	-	-	-	-	3.528 a	-	-	1.588 a	-	0.988	0.000
*4-aminobutyric acid*	-	-	-	-	0.393 a	1.122 a	-	-	-	0.193	0.000
*Threonic acid*	-	-	-	3.512 a	-	2.879 a	4.364 a	-	-	01364	0.145
*Lauric acid*	-	-	-	-	0.488 a	-	-	0.548 a	-	0.001	0.000
*Citric acid*	46.515 ab	56.955 ab	-	42.347 ab	-	-	-	27.936 a	-	10.955	0.005
*Ascorbic acid*	2.745 a		10.005 ab	4.495 a	43.774 bc	-	8.855 ab	24.466 b	82.472 c	4.954	0.000
Fatty acids											
*Palmitic acid*	7.131 ab	4.657 a	9.961 b	9.068 b	6.166 ab	8.678 ab	8.458 ab	8.165 ab	1.811 a	1.911	0.000
*Linoleic acid*	4.56 a	5.195 a	6.556 a	6.473 a	5.993 a	5.629 a	9.204 ab	9.397 ab	1.203 b	1.032	0.230
*a-linolenic acid*	-	-	-	-	2.492 a	-	-	4.541 a	0.675 a	0.025	0.050
*Estearic acid*	5.652 a	3.405 a	5.312 a	3.846 a	2.985 a	5.415 a	5.226 a	3.834 a	0.811 a	0.862	0.000
Phytosterols											0.000
γ-Tocopherol	0.531 a	3.274 b	2.132 ab	-	0.316 a	2.241 ab	1.238 ab	0.172 a	0.082 a	0.351	0.002
(*+*)-a-Tocopherol	-	0.609 ab	12.41 c	0.799 ab	2.298 b	13.129 c	8.75 bc	1.576 ab	0.335 a	0.309	0.000
*Campesterol*	3.037 ab	5.165 ab	5.971 ab	0.624 a	1.264 a	5.771 ab	4.196 ab	0.753 a	0.195 a	0.357	0.000
*Stigmasterol*	1.031 ab	2.845 b	1.438 ab	0.157 a	0.306 a	1.68 ab	1.209 ab	0.235 a	0.049	0.249	0.000
*β-Sitosterol*	9.478 ab	8.739 ab	17.216 b	1.758 a	2.412 a	16.887 b	10.763 ab	1.7 a	0.296 a	2.587	0.810

Results are expressed as μg of compounds per g of sweet pepper and are the average of three independent determinations ± SE. Different letters indicate significant statistical differences for each compound (*p < 0.05*, tukey’s test), P: probability levels.

**Table 2:** Treatments Applied to Sweet Pepper.

Treatments	SA (mM)	H_2_O_2_ (mM)	EC (dS/m)
1	0	0	3.2
2	0	36	3.2
3	0.1	0	3.2
4	0.1	36	3.2
5	0	0	3.6
6	0	36	3.6
7	0.1	0	3.6
8	0.1	36	3.6
9	0.01	18	3.4

## Data Availability

The data is found in the manuscript.
